# Relationship of short-term blood pressure variability with carotid intima-media thickness in hypertensive patients

**DOI:** 10.1186/s12938-015-0059-8

**Published:** 2015-07-24

**Authors:** Yujie Chen, Huahua Xiong, Dan Wu, Sandeep Pirbhulal, Xiaohong Tian, Ruiqin Zhang, Minhua Lu, Wanqing Wu, Wenhua Huang

**Affiliations:** Guangdong Provincial Key laboratory of Medical Biomechanics, Department of Anatomy, School of Basic Medical Science, Southern Medical University, Guangzhou, 510515 China; Department of Ultrasound, The Second People’s Hospital of Shenzhen, Shenzhen, 518029 China; Cardiac Electrocardiogram Room, The Second People’s Hospital of Shenzhen, Shenzhen, 518029 China; National-Regional Key Technology Engineering Laboratory of China for Medical Ultrasound, Department of Biomedical Engineering, Health Science Center, Shenzhen University, Shenzhen, 518060 China; Key Laboratory for Health Informatics, Chinese Academy of Sciences, Shenzhen, 518055 China; Shenzhen College of Advanced Technology, University of Chinese Academy of Sciences, Shenzhen, China; Institute of Biomedical and Health Engineering, Shenzhen Institutes of Advanced Technology, Chinese Academy of Sciences, Shenzhen, 518055 China

**Keywords:** Blood pressure variability, Intima-media, Hypertension

## Abstract

**Background:**

High blood pressure (BP) is among significant risk factor for stroke and other vascular occurrences, it experiences nonstop fluctuations over time as a result of a complex interface among cardiovascular control mechanisms. Large blood pressure variability (BPV) has been proved to be promising in providing potential regulatory mechanisms of the cardiovascular system. Although the previous studies also showed that BPV is associated with increased carotid intima-media thickness (IMT) and plaque, whether the correlation between variability in blood pressure and left common carotid artery-intima-media thickness (LCCA-IMT) is stronger than right common carotid artery-intima-media thickness (RCCA-IMT) remains uncertain in hypertension.

**Methods:**

We conduct a study (78 hypertensive subjects, aged 28–79) to evaluate the relationship between BPV and carotid intima-media thickness in Shenzhen. The blood pressure was collected using the 24 h ambulatory blood pressure monitoring, and its variability was evaluated using standard deviation (SD), coefficient of variation (CV), and average real variability (ARV) during 24 h, daytime and nighttime. All the IMT measurements are collected by ultrasound.

**Results:**

As the results showed, 24 h systolic blood pressure variability (SBPV) evaluated by SD and ARV were significantly related to LCCA-IMT (r^1^ = 0.261, P = 0.021; r^1^ = 0.262, P = 0.021, resp.). For the daytime diastolic blood pressure variability (DBPV), ARV indices were significantly related to LCCA-IMT (r^1^ = 0.239, P = 0.035), which differed form BPV evaluated by SD and CV. For the night time, there is no significant correlation between the BPV and IMT. Moreover, for all the subjects, there is no significant correlation between the BPV and RCCA-IMT/number of plaques, whereas, the SD, CV, and ARV of daytime SBP showed a positive correlation with LCCA-IMT (r^1^ = 0.312, P = 0.005; r^1^ = 0.255, P = 0.024; r^1^ = 0.284, P = 0.012, resp.). Moreover, the ARV of daytime SBPV, 24 h SBPV and nighttime DBPV showed a positive correlation with the number of plaques of LCCA (r^1^ = 0.356, P = 0.008; r^1^ = 0.297, P = 0.027; r^1^ = 0.278, P = 0.040, resp.). In addition, the number of plaques in LCCA had higher correlation with pulse pressure and diastolic blood pressure than that in RCCA. And multiple regression analysis indicated LCCA-IMT might not only be influenced by age or smoking but also by the SD index of daytime SBPV (p = 0.035).

**Conclusions:**

The results show that SBPV during daytime and 24 h had significant correlation with IMT, for the hypertensive subjects from the southern area of China. Moreover, we also found the daytime SBPV to be the best predictor for the progression of IMT in multivariate regression analysis. In addition, the present study suggests that the correlation between BPV and left common carotid artery—intima-media thickness/number of plaques is stronger than right common carotid artery-intima-media thickness/number of plaques.

## Background

High blood pressure (BP) is the most important risk factor for stroke [1–3] and other vascular events, accounting for approximately 54% of stroke and 47% of ischemic heart disease worldwide [4]. Physiologically, BP undergoes continuous fluctuations over time as a result of a complex interaction among cardiovascular control mechanisms. Recently, the study suggests that blood pressure variability (BPV) has been proved to be promising in providing potential regulatory mechanisms of the cardiovascular system [5]. Frattola et al. also constitute the first longitudinal evidence that cardiovascular complications of hypertension may depend on the degree of 24-h BPV [6]. Moreover, the population-based prospective studies [7, 8] have shown that ambulatory blood pressure (ABP) better predicts than clinic BP measurements the risk of subsequent cardiovascular events. So in our study the assessment of short-term BPV in the clinical setting is made possible by the growing use of ambulatory BP monitoring (ABPM) [7, 9]. From these recordings, it is possible to examine the prognostic of ABPM and its BPV evaluated with standard deviation (SD), coefficient of variation (CV), and average real variability (ARV) over the hypertensive people in the southern area of China.

In previous studies, in both longitudinal [6, 10–12] and cross-sectional studies [13–16], has clearly shown that useful information may arise from analysis of BPV, and variation in blood pressure has also been reported to be associated with cerebrovascular diseases. One cause link for this association was the development of atherosclerosis [11, 17]. And the widely use of B-mode ultrasound offers the opportunity to assess the intima-media thickness (IMT) of carotid artery as a reliable market for the extent of atherosclerosis [18, 19]. Date from previous investigations indicated that the carotid IMT was positively correlated to ambulatory blood pressure measurement (ABPM) [13, 14]. For example, in the study of Sander et al. [14], they found that the SD of diurnal systolic BPV as the strongest predictor for IMT. The previous research [15] also showed that both the daytime, and 24 h systolic BPV evaluated by SD, CV, and ARV are positively associated with IMT. Mena et al. [20] found that ARV added prognostic value to the ABPM [15] and could prompt the use of therapeutic measures to control BPV. Therefore the question that has arisen from the above findings is obviously which pressure has the greater clinical significance and can thus be taken as the best predictor of the patient’s cerebrovascular risk.

This paper will address this issue on the basis of the relationship between BPV and IMT to demonstrate the major impact of circadian blood pressure patterns on the development of early carotid atherosclerosis. In our study, we calculate SD, CV, and ARV of average systolic (SBP), diastolic (DBP), and mean BP values over the 24 h, with the daytime and the nighttime periods separately considered. We designed this study to prospectively analyze the relationship between changes in circadian blood pressure patterns and the progression of early carotid atherosclerosis. The aim is to found the greater association between IMT and BPV evaluated by three indices in hypertension, in the hope of promoting the application of 24-h BPV for the assessment of preclinical atherosclerosis.

## Methods

### Study population

The study was conducted in the Second People’s Hospital of Shenzhen, Guangdong Province, China. 78 individuals aged 28–79 years (57.7% male gender) were enrolled in this study. They fulfilled the following inclusion criteria: (1) patients with a clinical diagnosis of essential hypertension; (2) no history or clinical evidence of diabetes mellitus (fasting serum glucose <7.0 mmol/L; non fasting serum glucose <11.1 mmol/L); (3) both 24 h BP monitoring and carotid artery ultrasound measurement were performed; (4) the valid BP measurements within 24 h >90%. The Institutional Ethics Committee of the Second People’s Hospital of Shenzhen (China) approved this study, and the informed consent was obtained from every subject.

### Ambulatory BP measurement

All of the subjects underwent 24 h ABPM on a day of daily activity. A proper cuff was selected according to the size of subject’s arm and placed on the non-dominant arm. The subjects were asked to keep their arms still at the time of measurements. The ambulatory BP was recorded automatically using a commercial device (MobilGraph 24 h ABP-Control). The daytime BP monitoring was from 7:00 to 22:59, measured automatically every 30 min, and during the nighttime, from 23:00 to 6:59, the BP was measured once an hour. According to the recorded 24 h BP measurements, BPV was evaluated through the calculations of SD, CV, and ARV of the SBP and diastolic BP (DBP) during daytime, nighttime, and over 24 h. For the short-term (with 24 h) BPV analysis, SD, CV, and ARV are the common indices of BPV in time domain.

### Carotid artery ultrasound examination

The carotid artery ultrasound was examined using a high-resolution ultrasound Doppler system (iU22, Philips Ultrasound, Bothell, WA, USA), with a 7.5 MHz liner array transducer. During the examination, the subjects were supine in the bed, with the head turned 45° away from the examined side. The left and right common carotid arteries, carotid bulbs, and internal carotid arteries were scanned in three angles (lateral, anterior, and posterior). Thus, we can assess the mean IMT in each position from the three measurements in different angles. The specific places we measured in the carotid artery were defined as follows: the IMT at the common carotid artery was measured on the far wall of blood vessel, 10–20 mm proximal to the carotid bifurcation. The carotid bulb we measured was in the carotid bifurcation, and the IMT at the internal carotid artery was measured over a distance of 10–20 mm from the bifurcation. In our study, the correlation analysis will focus on the common carotid artery, and thus carotid IMT in this paper represents the IMT at the common carotid artery, which is an average of right and left IMT. Besides, the abnormal IMT is defined that the IMT at the common carotid artery is more than 1.0 mm.

### Statistical analysis

All statistical analyses were performed using SPSS statistical package (SPSS Inc., Chicago, IL, USA). Simple regression analyses for BPV and IMT/number of plaques were determined. Quintile analysis was applied to determine the relation between BPV and IMT, in which subjects were divided into five groups according to the distribution of the variability. Multiple regression analyses were also performed to evaluate the relation between BPV and IMT. We defined the carotid IMT/number of plaques as the dependent factor and the BPV estimated with SD, CV, and ARV as the independent factors, respectively. When the correlation coefficient r was close to 1, it indicated that the BPV had highly positive correlation with IMT. On the contrary, when r was close to −1, the relativity about BPV and IMT was negative. A P value of <0.05 was considered significant. Data are reported as mean ± SD.

## Results

Among all of the hypertensive participants, we excluded the cases that had the incomplete or invalid measurements. Finally, a total of 78 patients aged 28–79 (male 57.7%) were successfully obtained in the study. Of those, 43 subjects had the normal carotid IMT, and 35 subjects had a carotid IMT more than 1.0 mm, which is defined as the abnormal IMT. Table 1 summarized the clinical characteristics of all the subjects and two subgroups: the subjects with the normal IMT and the subjects with the abnormal IMT. The data of clinical characteristics were expressed as means ± SDs or percentages. In this table, mean SBP and DBP in different periods of time, mean PP in daytime and nighttime, BP decreasing percent from day to night, IMTs at different carotid arteries and the plaque status were reported. No significant differences were documented between the normal IMT group and abnormal IMT group regarding the BP values. However, for the baseline characteristics, the subjects in the abnormal IMT group were significantly older than the subjects in the normal IMT group (P < 0.05). And smoking rates in the abnormal IMT group was higher (34.3%), which is statistical significance (P < 0.05). Besides, in the abnormal IMT group, 77.1% of subjects had the plaques, which is higher than that in the normal IMT group (P < 0.001). Moreover, the abnormal IMT group had a significantly greater IMT both at bulb and internal carotid artery than the normal IMT group (P < 0.001), and most of them tended to suffer from the prevalence of the atherosclerotic plaques (P < 0.001). We evaluated the BPV using SD, CV, and ARV, and the average BPV values quantified with three indices in 24 h were reported in Table 2. We compared the correlations of these BPV values in each group of the two using Pearson’s test. Except the DBPV in nighttime between CV and SD, ARV respectively. (P > 0.05), no significant differences were found among the three indices of BPV, they had strongly positive correlation (P < 0.01). Moreover, we found that all of the systolic blood pressure variability (SBPV) values were greater than those of DBPV when evaluated using SD and ARV. In contrast, the DBPV values were found to be greater than SBPV when using CV as a measure.

**Table 1:** Clinical characteristics of all the subjects and the two subgroups: normal IMT group and abnormal IMT group

Characteristics	All subjects (N = 78)	IMT <1.0 mm (N = 43)	IMT ≥1.0 (N = 35)	T(x^2^)值	P value
Age (years)	55.9 ± 13.0	51.7 ± 12.9	61.1 ± 11.4	−3.374	0.001**
Male gender (%)	57.7	55.8	60	−0.368	0.714
Smoking (%)	23.1	14	34.3	−2.088	0.041*
IMT ≥1.0 mm	44.9	0	100	−9.950	0.000**
Presence of plaque (%)	51.3	30.2	77.1	−4.601	0.000**
CCA IMT (mm)	1.0 ± 0.2	0.78 ± 0.12	1.16 ± 0.11	−14.474	0.000**
Bulb IMT (mm)	0.8 ± 0.3	0.67 ± 0.14	1.00 ± 0.26	−6.754	0.000**
ICA IMT (mm)	0.6 ± 0.1	0.52 ± 0.08	0.66 ± 0.13	−5.630	0.000**
24 h SBP (mmHg)	125.1 ± 15.4	124.5 ± 13.4	125.9 ± 17.7	−0.394	0.695
24 h DBP (mmHg)	81.5 ± 12.8	82.7 ± 11.8	80.0 ± 14.0	0.928	0.356
24 h PP (mmHg)	43.6 ± 8.6	41.8 ± 6.4	45.9 ± 10.5	−2.030	0.047*
Daytime SBP (mmHg)	126.2 ± 15.3	125.5 ± 13.2	127.0 ± 17.7	−0.430	0.669
Daytime DBP (mmHg)	84.3 ± 15.8	84.8 ± 12.9	83.7 ± 18.9	0.284	0.777
Daytime PP (mmHg)	41.9 ± 11.3	40.7 ± 8.4	43.4 ± 14.1	−0.973	0.335
Nighttime SBP (mmHg)	121.0 ± 17.6	120.4 ± 16.0	121.6 ± 19.6	−0.298	0.767
Nighttime DBP (mmHg)	77.6 ± 13.7	78.3 ± 12.8	76.8 ± 14.9	0.484	0.630
Nighttime PP (mmHg)	43.3 ± 10.0	42.1 ± 7.8	44.8 ± 12.2	−1.109	0.272
SBP decrease (%)	2.3 ± 6.8	2.7 ± 5.2	1.9 ± 8.2	0.371	0.713
DBP decrease (%)	4.1 ± 7.2	4.2 ± 5.4	4.0 ± 8.6	0.102	0.919

**Table 2:** The blood pressure variabilities evaluated with SD, CV, and ARV for all subjects (N = 78)

Variables	SD (mmHg)		CV (%)		ARV (mmHg)	
	Mean ± SD	r(P)^1^	Mean ± SD	r(P)^2^	Mean ± SD	r(P)^3^
Daytime SBPV	11.2 ± 3.5	0.865** (0.000)	9.2 ± 2.5	0.687** (0.000)	9.1 ± 2.7	0.801** (0.000)
Daytime DBPV	9.1 ± 2.7	0.286* (0.011)	13.4 ± 4.1	0.497** (0.000)	9.1 ± 2.9	0.623** (0.000)
Nighttime SBPV	9.5 ± 3.6	0.377** (0.001)	9.3 ± 3.0	0.377** (0.001)	10.1 ± 4.6	0.861** (0.000)
Nighttime DBPV	8.0 ± 2.6	0.164 (0.152)	14.4 ± 4.6	0.093 (0.420)	8.9 ± 2.9	0.768** (0.000)
24 h SBPV	11.6 ± 3.2	0.891** (0.000)	9.3 ± 2.6	0.731** (0.000)	9.1 ± 2.7	0.823** (0.000)
24 h DBPV	9.2 ± 2.0	0.453** (0.000)	13.9 ± 4.0	0.439** (0.000)	8.0 ± 1.9	0.662** (0.000)

Table 3 depicted the correlation coefficients between different indices of BPV and carotid IMT in all subjects. As the results showed, for all the subjects, there is no significant correlation between the BPV and RCCA-IMT, whereas, the SD, CV, and ARV of daytime SBP showed a positive correlation with LCCA-IMT (r^1^ = 0.312, P = 0.005; r^1^ = 0.255, P = 0.024; r^1^ = 0.284, P = 0.012, resp.). Moreover, 24 h SBPV evaluated by SD and ARV were significantly related to LCCA-IMT (r^1^ = 0.261, P = 0.021; r^1^ = 0.262, P = 0.021, resp.). For the daytime DBPV, ARV indices was significantly related to LCCA-IMT (r^1^ = 0.239, P = 0.035), which differed form BPV evaluated by SD and CV. For the night time, there is no significant correlation between the BPV and IMT. In addition, for the mean CCA-IMT, only daytime SBPV evaluated with SD and ARV indices were significant (for SD, r^3^ = 0.231, P = 0.041; for ARV, r^3^ = 0.266, P = 0.019.). In addition, the correlations between the different indices of BPV and number of plaques also were shown in Table 3. The results indicated that there was no significant correlation between BPV and the number of plaques of RCCA, whereas, the ARV of daytime SBPV, 24 h SBPV and nighttime DBPV showed a positive correlation with the number of plaques of LCCA (r^1^ = 0.356, P = 0.008; r^1^ = 0.297, P = 0.027; r^1^ = 0.278, P = 0.040, resp.). Moreover, for the number of plaques of mean CCA, only daytime SBPV evaluated with ARV was significant (r^3^ = 0.278, P = 0.016).

**Table 3:** The correlation between the blood pressure variability (evaluated with SD, CV, and ARV) and carotid intima**-**media thickness/number of plaques in all the subjects

Variables	IMT			Number of plaques		
	r(P)^1^	r(P)^2^	r(P)^3^	r(P)^1^	r(P)^2^	r(P)^3^
SD						
Daytime SBPV	0.312** (0.005)	0.103 (0.368)	0.231* (0.041)	0.146 (0.286)	0.073 (0.596)	0.065 (0.584)
Daytime DBPV	0.098 (0.391)	0.037 (0.745)	0.076 (0.51)	0.002 (0.989)	−0.105 (0.443)	−0.104 (0.378)
Nighttime SBPV	0.053 (0.646)	0.008 (0.945)	0.033 (0.771)	0.156 (0.255)	0.078 (0.572)	0.014 (0.907)
Nighttime DBPV	0.046 (0.686)	0.136 (0.235)	0.105 (0.359)	0.215 (0.115)	0.149 (0.279)	0.142 (0.227)
24 h SBPV	0.261* (0.021)	0.119 (0.298)	0.213 (0.061)	0.151 (0.272)	0.100 (0.467)	0.065 (0.584)
24 h DBPV	0.098 (0.392)	0.068 (0.555)	0.093 (0.416)	−0.005 (0.970)	−0.058 (0.671)	−0.104 (0.378)
CV						
Daytime SBPV	0.255* (0.024)	0.117 (0.306)	0.208 (0.067)	0.167 (0.223)	0.122 (0.373)	0.105 (0.374)
Daytime DBPV	0.172 (0.132)	0.132 (0.248)	0.172 (0.133)	0.174 (0.203)	0.077 (0.575)	0.097 (0.410)
Nighttime SBPV	0.08 (0.485)	0.146 (0.201)	0.13 (0.258)	0.097 (0.481)	0.080 (0.560)	0.059 (0.616)
Nighttime DBPV	0.007 (0.95)	0.143 (0.212)	0.088 (0.444)	0.172 (0.209)	0.195 (0.153)	0.130 (0.268)
24 h SBPV	0.215 (0.059)	0.146 (0.202)	0.203 (0.075)	0.175 (0.202)	0.142 (0.301)	0.122 (0.301)
24 h DBPV	0.145 (0.206)	0.153 (0.18)	0.169 (0.139)	0.200 (0.142)	0.145 (0.292)	0.118 (0.316)
ARV						
Daytime SBPV	0.284* (0.012)	0.188 (0.099)	0.266* (0.019)	0.356** (0.008)	0.241 (0.077)	0.278* (0.016)
Daytime DBPV	0.239* (0.035)	0.076 (0.511)	0.175 (0.126)	0.150 (0.275)	0.100 (0.467)	0.076 (0.523)
Nighttime SBPV	−0.014 (0.904)	−0.008 (0.941)	−0.013 (0.913)	0.155 (0.259)	0.054 (0.698)	0.025 (0.834)
Nighttime DBPV	0.053 (0.646)	0.16 (0.162)	0.123 (0.285)	0.278* (0.040)	0.137 (0.317)	0.186 (0.113)
24 h SBPV	0.262* (0.021)	0.122 (0.286)	0.215 (0.059)	0.297* (0.027)	0.151 (0.270)	0.188 (0.108)
24 h DBPV	0.202 (0.077)	0.097 (0.398)	0.167 (0.143)	0.241 (0.077)	0.129 (0.348)	0.138 (0.240)

**Fig. 1:**
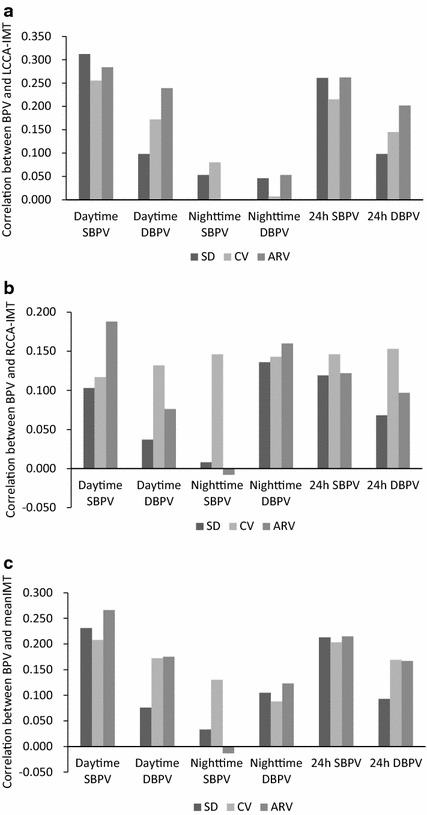
The correlation of the blood pressure variability and carotid intima-media thickness in all subjects. *BPV* blood pressure variability, *IMT* intima-media thickness, *CV* coefficient of variation, *ARV* average real variability, *SBPV* systolic blood pressure variability, *DBPV* diastolic blood pressure variability.

As Fig. 1 showed, the SBPV during daytime and 24 h had greater correlation than DBPV during daytime and 24 h. Moreover, the correlations of the SBPV (evaluated with SD, CV, and AVR) and IMT were almost the same. However, for the DBPV during the daytime and 24 h, the SD and CV indices of BPV had greater correlation with IMT than ARV index.

**Fig. 2:**
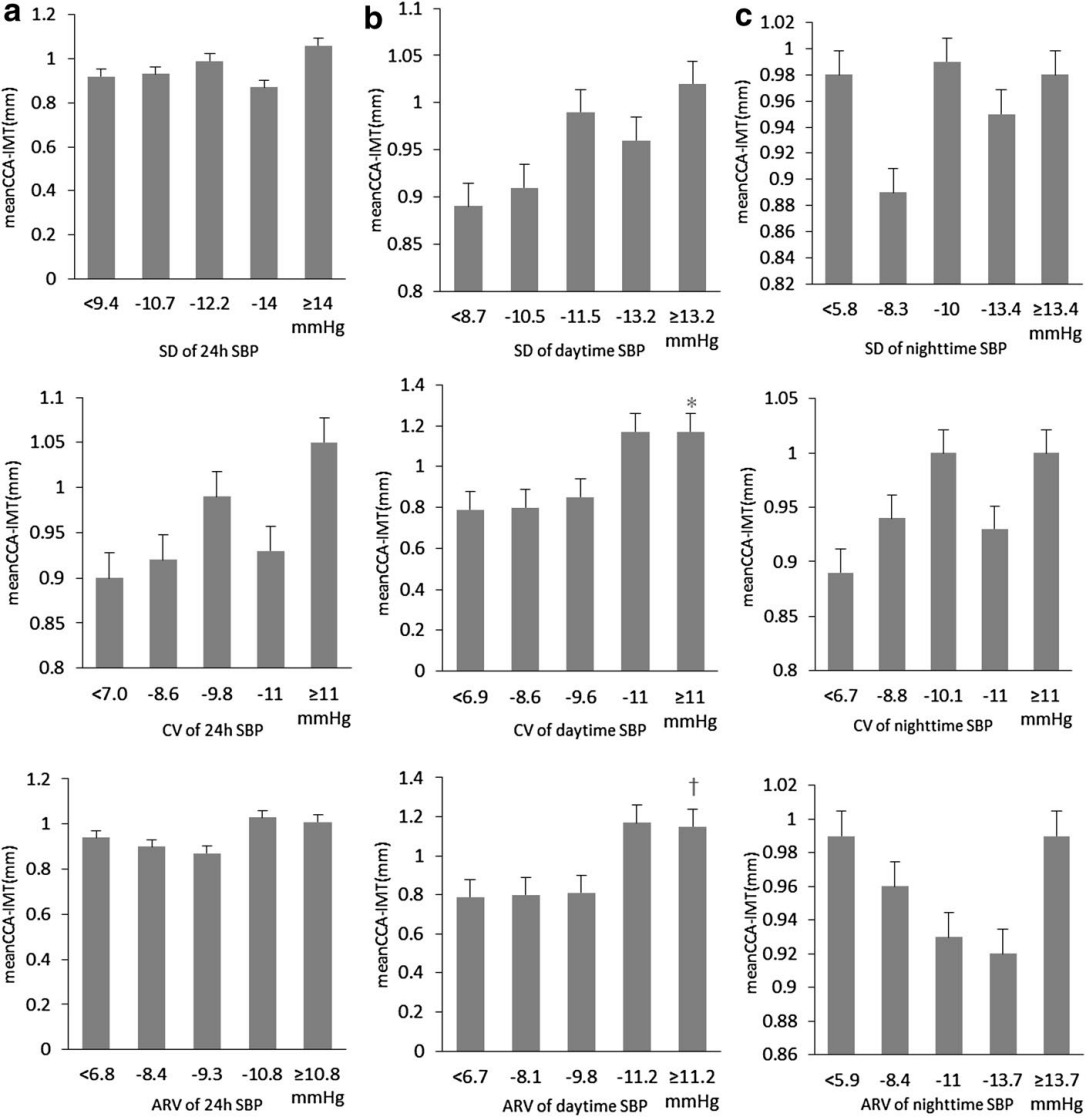
Changes in mean CCA-IMT in association with stepwise increases in SBPV (evaluated with SD, CV, and ARV) in hypertensives. Values are means ± S.E.M. **a** Variability of 24 h SBP. **b** Variability of daytime SBP. *P < 0.05 vs the <6.9 mmHg group. ^†^P < 0.05 vs the <8.1 mmHg group. **c** Variability of nighttime SBP.

To further compare the results, we described these correlations between variability of SBP and mean CCA-IMT in Fig. 2. It assesses association of increases in mean CCA-IMT with stepwise increases in the variability of 24-h, daytime and nighttime SBP (evaluated with SD, CV, and ARV). The correlation of three indices of SBPV during daytime and mean CCA-IMT were almost the same. Moreover, the mean CCA-IMT of patients who’s SD of daytime SBP was 10.5 mmHg and above was significantly higher than that of the patients whose SD of daytime SBP was below 10.5 mmHg.

The mean CCA-IMT of patients having CV of daytime SBP was 9.6 mmHg and above was significantly higher than that of the patients who are CV of daytime SBP was below 9.6 mmHg. The mean CCA-IMT of patients who’s ARV of daytime SBP was 9.8 mmHg and above was significantly higher than that of the patients who are ARV of daytime SBP was below 9.8 mmHg. However, for the increases in 24 h and nighttime SBPV (evaluated with SD, CV, and ARV respectively) showed different mean CCA-IMT values.

The correlations between the average BP values and carotid IMT/number of plaques were analyzed using Pearson’s test. The results were shown in Table 4. It indicated that there was no significant correlation between these BP variables and carotid IMT, whereas, 24 h PP, daytime PP, and nighttime PP were positively associated with the number of plaques (for LCCA, r^1^ = 0.485, P^1^ = 0.000; r^1^ = 0.465, P^1^ = 0.000; r^1^ = 0.510, P^1^ = 0.000, resp.; for RCCA, r^2^ = 0.394, P^2^ = 0.003; r^2^ = 0.375, P^2^ = 0.005; r^2^ = 0.413, P^2^ = 0.002, resp.; for mean CCA, r^3^ = 0.358, P^3^ = 0.002; r^3^ = 0.353, P^3^ = 0.002; r^3^ = 0.374, P^3^ = 0.001, resp.). Moreover, a negative correlation was found between the nighttime average DBP and the number of plaques (for LCCA, r^1^ = −0.443, P^1^ = 0.001; r^1^ = −0.463, P^1^ = 0.000; r^1^ = −0.339, P^1^ = 0.011, resp.; for RCCA, r^2^ = −0.412, P^2^ = 0.002; r^2^ = −0.416, P^2^ = 0.002; r^2^ = −0.369, P^2^ = 0.006, resp.; for mean CCA, r^3^ = −0.372, P^3^ = 0.001; r^3^ = −0.391, P^3^ = 0.001; r^3^ = −0.308, P^3^ = 0.008, resp.). In addition, the number of plaques in LCCA had higher correlation with PP and DBP than that in RCCA.

**Table 4:** The correlation between mean blood pressure values and carotid intima**-**media thickness/number of plaques in all the subjects

Variables	IMTc			Number of plaques		
	r(P)^1^	r(P)^2^	r(P)^3^	r(P)^1^	r(P)^2^	r(P)^3^
24 h SBP	0.073 (0.525)	−0.048 (0.673)	0.011 (0.921)	−0.083 (0.548)	−0.111 (0.420)	−0.109 (0.356)
24 h DBP	−0.033 (0.776)	−0.190 (0.096)	−0.129 (0.259)	−0.443** (0.001)	−0.412** (0.002)	−0.372** (0.001)
24 h PP	0.177 (0.120)	0.194 (0.089)	0.211 (0.064)	0.485** (0.000)	0.394** (0.003)	0.358** (0.002)
Daytime SBP	0.059 (0.606)	−0.040 (0.725)	0.009 (0.940)	−0.122 (0.373)	−0.132 (0.338)	−0.135 (0.251)
Daytime DBP	−0.059 (0.610)	−0.167 (0.145)	−0.130 (0.257)	−0.463** (0.000)	−0.416** (0.002)	−0.391** (0.001)
Daytime PP	0.161 (0.159)	0.176 (0.123)	0.191 (0.093)	0.465** (0.000)	0.375** (0.005)	0.353** (0.002)
Nighttime SBP	0.100 (0.382)	−0.059 (0.605)	0.020 (0.086)	0.036 (0.795)	−0.046 (0.738)	−0.025 (0.833)
Nighttime DBP	0.045 (0.699)	−0.160 (0.161)	−0.170 (0.544)	−0.339* (0.011)	−0.369** (0.006)	−0.308** (0.008)
Nighttime PP	0.113 (0.324)	0.118 (0.303)	0.131 (0.252)	0.510** (0.000)	0.413** (0.002)	0.374** (0.001)
SBP decrease (%)	−0.062 (0.702)	−0.087 (0.590)	−0.087 (0.588)	−0.200 (0.216)	−0.127 (0.434)	−0.169 (0.299)
DBP decrease (%)	−0.064 (0.690)	0.029 (0.856)	−0.021 (0.895)	−0.129 (0.428)	0.024 (0.882)	−0.031 (0.849)

To further compare the effects of different indices of BPV on LCCA-IMT, Multiple regression analysis for 4 independent factors indicated significant correlations between LCCA-IMT and age and smoking in Table 5. Thus, LCCA-IMT may not only be influenced by age or smoking but also by the SD index of daytime SBPV. However, LCCA-IMT was not correlated with any other ambulatory BPVs.

**Table 5:** Multiple regression analysis of left carotid intima-media thickness and blood pressure variabilities

Factors	β (95% CI)	P value	R^2^
Age	0.006 (0.002 to 0.011)	0.005	0.296
Smoking	0.220 (0.104 to 0.336)	0.000	
Hypertension duration	0.002 (−0.004 to 0.008)	0.440	
24 h SBPV (SD)	0.008 (−0.008 to 0.024)	0.324	
Age	0.006 (0.002 to 0.010)	0.007	0.330
Smoking	0.222 (0.110 to 0.029)	0.000	
Hypertension duration	0.002 (−0.003 to 0.008)	0.400	
daytime SBPV (SD)	0.015 (0.001 to 0.029)	0.035	
Age	0.006 (0.002 to 0.010)	0.007	0.307
Smoking	0.229 (0.116 to 0.342)	0.000	
Hypertension duration	0.003 (−0.003 to 0.008)	0.399	
Daytime SBPV (CV)	0.014 (−0.006 to 0.034)	0.154	
Age	0.006 (0.002 to 0.011)	0.007	0.292
Smoking	0.222 (0.105 to 0.338)	0.000	
Hypertension duration	0.002 (−0.004 to 0.008)	0.426	
24 h SBPV (ARV)	0.007 (−0.012 to 0.026)	0.457	
Age	0.006 (0.002 to 0.010)	0.006	0.298
Smoking	0.218 (0.102 to 0.334)	0.000	
Hypertension duration	0.002 (−0.004 to 0.008)	0.457	
Daytime SBPV (ARV)	0.009 (−0.008 to 0.027)	0.283	
Age	0.007 (0.002 to 0.011)	0.002	0.291
Smoking	0.217 (0.095 to 0.339)	0.001	
Hypertension duration	0.002 (−0.004 to 0.008)	0.452	
Daytime DBPV (ARV)	0.007 (−0.016 to 0.030)	0.522	

## Discussion

The results of the present study showed that the SBP fluctuations during daytime and 24 h were significantly associated with the increased carotid IMT. Moreover, for all the subjects, there is no significant correlation between the BPV and right CCA-IMT/number of plaques, whereas, the SD, CV, and ARV of daytime SBP showed a positive correlation with LCCA-IMT. Moreover, the ARV of daytime SBPV, 24 h SBPV and nighttime DBPV showed a positive correlation with the number of plaques of LCCA. In addition, quintile stepwise analyses showed that the correlation of three indices of SBPV during daytime and mean CCA-IMT were almost the same, and the carotid IMT showed a progressively greater value from the quintile with the lowest to the quintile with the highest daytime SBPVs. But it did not show any noticeable or consistent variation from the quintile with the lowest to the quartile with the highest 24 h or nighttime SBPVs. For further multiple regression analysis, we found that the daytime SBPV evaluated with SD was significantly associated with carotid IMT.

In earlier studies, BP fluctuations being the result of a complex interaction between environmental stimuli and the response of cardiovascular control mechanisms [21–23]. BPV is characterized by marked short-term BPV occurring within a 24-h period (beat-to-beat, minute-to-minute, hour-to-hour, and day-to-night changes) and also by long-term BPV occurring over more-prolonged periods of time (days, weeks, months, seasons, and even years) [24]. Compared with long-term BPV, short-term BPV indicators were easier to measure and collect. Previous studies also demonstrated that good control of ambulatory BP has a more beneficial effect on cardiovascular organ damage in hypertensive patients than good control of clinic BP [25], and reported that the association between short-term BPV derived from 24-h ABPM and carotid IMT [11, 13, 15, 26–29]. Thus, tight BP control throughout the 24-h period structurally and functionally improves the stiffened arterial walls of hypertensive patients.

Our results corroborate previous findings. Firstly, in the earlier study [15], it was reports that the relationship between the 24 h BPV and carotid IMT. They found that both the daytime, and 24 h systolic BPV evaluated by SD, CV, and ARV are positively associated with IMT (for daytime SBPV, r = 0.408, P = 0.001; r = 0.381, P = 0.003; r = 0.396, P = 0.002, resp.; for 24 h SBPV, r = 0.339, P = 0.002; r = 0.376, P = 0.003; r = 0.339, P = 0.008, resp.). They also found the relationship between carotid IMT and DBPV during the daytime and 24 h, the SD and CV indices of BPV had greater correlation than ARV index (for daytime DBPV, r = 0.293, P = 0.023; r = 0.302, P = 0.019, resp.; for 24 h DBPV, r = 0.328, P = 0.010; r = 0.323, P = 0.012, resp.). The similar conclusions were proposed from the study of Sander et al. [11]. They indicated that the progression of IMT was significantly greater in the patients with increased SBPV, multivariate regression analysis also revealed the daytime SBPV to be the best predictor for the progression of IMT. Our study presents new evidence because the previous demonstrations of its significant conclusion have been derived mainly from population-based study [15] rather than hypertensive patients. We also suggested that the results of correlation analysis for the relationship between SBPV and IMT in hypertensive patient were consistent with their studies.

Secondly, previous studies mostly focused on exploring the association between 24 h ambulatory BP variability and mean carotid IMT [11, 15], and comparing the effects on the carotid artery structure for different indices of BPV [20, 30]. In the earlier study [15], they found that daytime systolic BPV evaluated with ARV is the best variable to represent the increasing of carotid IMT. The similar conclusions have been show in the study of Mena et al. [20] and Hansen et al. [30]. Mena et al. found that the commonly used SD index may be more sensitive to the sampling frequency of the ABPM devices, and ARV index (RR = 1.611, P = 0.004) is a more reliable representation of time series variability than SD (RR = 1.103, P = 0.571) for the prognostic significant BPV. Hansen et al. also suggested that BPV was a significant and independent predictor of mortality and of cardiovascular and stroke events, ARV24 was a better predictor than SD24 and SDdn. Thus, ARV24 might be a more specific measure of BPV than SD. In our present study, we not only found that for the daytime DBPV evaluated with ARV was significantly related to LCCA-IMT (r^1^ = 0.239, P = 0.035), which differed form BPV evaluated by SD and CV, but also there is no significant correlation between the BPV and RCCA-IMT, whereas, the SD, CV, and ARV of daytime SBP showed a positive correlation with LCCA-IMT. Moreover, the present study indicated that there was no significant correlation between these BP variables and carotid IMT, whereas, the ARV of daytime SBPV, 24 h SBPV and nighttime DBPV showed a positive correlation with the number of plaques of LCCA. So the results also show that ABP better predicts than clinic BP measurements the risk of subsequent cardiovascular events as before studies [7, 8]. Moreover, 24 h PP, daytime PP, and nighttime PP were positively associated with the number of plaques in hypertensive patients. And a negative correlation was found between the nighttime average DBP and the number of plaques. The similar conclusions were proposed from the earlier study [15]. In addition, the number of plaques in LCCA had higher correlation with PP, DBP, SBPV and DBPV than that in RCCA. Despite this, more evidence is still required to assess whether the correlation between BPV and LCCA-IMT/number of plaques is stronger than the right.

Thirdly, we found that the correlation of three indices of SBPV during daytime and mean CCA-IMT were almost the same. Moreover, the maximum IMT increased progressively from the quintile with the lowest to the quintile with the highest daytime BPV evaluated with CV. However, for the increases in 24 h and nighttime SBPV (evaluated with SD, CV, and ARV respectively) showed different growing trends with mean CCA-IMT values, especially for the nighttime SBPV. In the earlier study, Mancia et al. showed that the end-of-treatment carotid CBM_max_ increased progressively and significantly from the quartile with the lowest to the quartile with the highest on-treatment 24-h SBP mean. But carotid CBM_max_ showed no significant difference between quartiles of on-treatment 24-h SBP CV or SD [31]. In our present study, we also did not find any significant difference between quintiles of SDs, CVs or ARVs in hypertensive patients. This study demonstrated growing trends between mean CCA-IMT and BPV evaluated with SD, CV and ARV.

Besides, other findings of our study also deserve to be discussed. The earlier study [15] suggested that no significant differences were found among the three indices of BPV when compared the correlations of these BPV values in each group of two using Pearson’s test, they had strongly positive correlation (P < 0.01). In our study, we did not find that the DBPV in nighttime showed positive correlation between CV and SD, ARV in hypertensive patients (P > 0.05). Moreover, in our multiple regression analysis, it showed that the correlation between daytime SBP evaluated with SD and the increased carotid IMT independent of the well-known confounding factors, such as age, smoking. Although the previous outcome-based studies which showed the superiority of night-time over daytime ambulatory BP averages for prediction of a composite pool of cardiovascular events [32, 33]. Kikuya et al. also demonstrated that an excessive BP variability, evaluated by an increased SD of night-time SBP, adds prognostic information to that provided by a wide ambulatory PP [34]. Our finding was consistent with the study of Sander et al. [14], they found that the SD of diurnal SBPV as the strongest predictor for IMT. The previous research [15] also showed that both the daytime, and 24 h systolic BPV evaluated by SD, CV, and ARV are positively associated with IMT.

Finally, certain limitations of the present study should be acknowledged. First, in our study, the BPV during the night time was not associated with IMT. Because in the present study, BP variability was measured as the SD, CV, and ARV of BP measurements every 30 min during the daytime and every 60 min during the night time. Time frequency of BP measurements is important at the time to estimate variability from non-invasive ABPM techniques. Therefore, the results of the present study should be confirmed by ambulatory BP measurements at a shorter interval. Second, both the relatively small size of the subjects and the cross-sectional survey are the important limitations in our present study. So the link between ambulatory BP variability and carotid atherosclerosis is unable to deduce a causal sequential conclusion. Third, the correlation between mean CCA-IMT and DBP variability evaluated with SD, CV and ARV were not demonstrated in present study. This issue remains to be addressed in future studies. In conclusion, though the limitations exist in our study, we also obtained the suggestive and significant conclusions. All important abbreviation used in this research paper are mentioned above.

## Conclusions

Our study provides the evidence that the SBPV during daytime and 24 h had significant correlation with IMT, for the hypertensive subjects from the southern area of China. Moreover, we also found the daytime SBPV to be the best predictor for the progression of IMT in multivariate regression analysis. In addition, the present study suggests that the correlation between BPV and LCCA-IMT/number of plaques is stronger than the right. However, more evidence is still required to assess whether the correlation between BPV and LCCA-IMT/number of plaques is stronger than the right in future studies. We also will conduct the large-scale trials and perform more analysis to investigate how to predict the risks of cardiovascular disease and mortality from the alteration of carotid structure and function.

## Abbreviations

BPV: blood pressure variability
IMT: intima-media thickness
LCCA: left carotid common artery
RCCA: right carotid common artery
BP: blood pressure
SD: standard deviation
CV: coefficient of variation
ARV: average real variability
SBPV: systolic BPV
DBPV: diastolic BPV
ABP: ambulatory blood pressure
ABPM: ambulatory BP monitoring
DBP: diastolic blood pressure
SBP: systolic blood pressure
ICA: internal carotid artery
CCA: carotid common artery
PP: pulse pressure
CI: confidence interval
